# Increased bacterial load of *Filifactor alocis* in deep periodontal pockets discriminate between periodontitis stage 3 and 4

**DOI:** 10.3389/froh.2025.1543030

**Published:** 2025-03-27

**Authors:** Reem H. Faisal, Alaa O. Ali

**Affiliations:** Department of Periodontics, College of Dentistry, University of Baghdad, Baghdad, Iraq

**Keywords:** periodontitis, periodontal disease, biofilm, dental biofilm, *Filifactor alocis*, attachment loss

## Abstract

**Introduction:**

Increasing evidence supports the association of *Filifactor alocis* with periodontitis; therefore, this work was conducted to assess the prevalence and proportion of *F. alocis* in subgingival biofilm samples from patients with periodontitis stage 3 and 4, and its potential to differentiate between these stages.

**Methods:**

This cross-sectional study included 50 periodontitis patients from whom subgingival biofilm samples were collected using paper points. This was followed by recording clinical periodontal parameters including the plaque index, bleeding on probing, probing pocket depth (PPD), and clinical attachment loss (CAL). The total loads of bacteria and *F. alocis* were determined via quantitative PCR.

**Results:**

All patients were diagnosed with periodontitis stage 3/4 and grade B/C, with a total of 727 periodontal pockets, which were pooled (*n* = 114) for microbiological analysis. Qualitative and quantitative analyses indicated that the total bacterial load and prevalence of *F. alocis* were highest in stage 4 and grade C cases, which were also increased with increasing PPD and severity of CAL. An ROC analysis indicated that both the total bacterial load and *F. alocis* concentration could significantly discriminate stage 3 and 4 periodontitis. The regression model suggested that a one-unit increase in PPD, and CAL could explain a 23.9% and 14.9% increase in the *F. alocis* concentration, respectively.

**Conclusion:**

The results demonstrate that the prevalence of *F. alocis* is increased in severe periodontitis cases, mainly at sites with deep periodontal pockets and greater attachment loss. Additionally, this bacterium possesses the diagnostic potential to differentiate periodontitis cases of different severities.

## 1 Introduction

Periodontitis is a multi-etiological and highly prevalent chronic inflammatory disease that affects the anchoring apparatus of the teeth which is mostly overlooked by the patients (1–3). The hallmark of this disease is the inflammation-associated collateral destruction of periodontal tissue, which is reflected by radiographic evidence of alveolar bone loss and clinical attachment loss (CAL). The latter two parameters are considered the cornerstone for the diagnosis of periodontitis according to 2017 classification of periodontal diseases which was outlines by the American Academy of Periodontology (AAP) and European Federation of Periodontolgy (EFP) (4). Combination of CAL measured at the worst site with radiographs aid in determining the severity i.e., stage by measuring the amount of bone loss relative to the length of the root. The result of dividing the percent of bone loss in the same site by patient's age helps in predicting the rate of future bone loss which is known as grade. According to this formula, the higher the number, the worst expected prognosis which aids in customizing the treatment for each patient (4). Later, the joint AAP/EFP workshop introduced for the first-time clear treatment guidelines for treatment of periodontitis based on the severity of disease. These guideline were published in two separate papers, the first outlined treatment steps and recommendation for periodontitis stage 1–3 (5), while the second one was dedicated for comprehensive detailing for multidisciplinary treatment options for periodontitis stage 4 (6). For all cases of periodontitis, diagnosis is the first step that must be accurately determined before commencing any treatment. Periodontal charting of the clinical parameters by using periodontal probe aided by radiograph is the gold standard and most widely used methods globally for diagnosis of periodontal disease. Although these techniques are relatively cheap, affordable, and easy to use, certain inherited limitations may compromise the results leading to a potential misdiagnosis; subsequently, improper treatment could be selected. Of these, relatively lengthy and tedious charting procedure, discomfort to the patient, manual dexterity of the clinician, size/dimension of the probe, force applied, and degree of angulation during measurement. Additionally, radiograph as an indispensable diagnostic tool, it reflects 2D image of a 3D object(s) that may obscure some landmarks and disease-related changes. Further, evidence of bone loss is not apparent on x-ray film until loss of mineral content reached over 30%. These limitations encouraged the search of efficient surrogates to predict, diagnose, monitor periodontal disease. In this respect, serious attempts are constantly growing to use host-derived and bacterial biomarkers as diagnostic tools.

The onset and progression of periodontal disease have always been attributed to the interaction between an aberrant host's immune response and bacteria in the dental plaque biofilm, which stand as the primary etiological factor (7, 8). The deleterious effect of periodontitis is not exclusive to periodontal tissues but extends systemically and negatively modulates a range of systemic diseases and conditions (9–11). The oral microbiome comprises nearly 700 species that physically and metabolically interact with one another to form unique and complex biofilm communities that provide an outstanding microenvironment for a wide range of bacteria with different metabolic requirements to thrive (12–14). Biofilm formation starts with the attachment of primary colonizers such as *Actinomyces spp.* and *Streptococcus spp.* supragingivally, providing a foundation for the attachment of other bacteria, such as *Corynebacterium spp*., on which further bacterial species can anchor (15). The increasing mass and complexity of the dental biofilm create an anoxic environment at its center, which is ideal for the growth of capnophilic species such as *Capnocytophaga* and *Fusobacterium*, which pave the way for the emergence of putative periodontal pathogens (15).

Dysbiosis is defined as a shift in the health-associated microbial populations or imbalance in the ecosystem of microorganisms that leads to a disruption of the advantageous interaction with the host (12). Consequently, dysbiotic dental biofilm compromises health, tipping the balance towards periodontal disease (16, 17). Hajishengallis et al. hypothesized that this shift in the bacterial population could be translated in two ways: First, it could indicate that the disease is initiated by dysbiosis of the periodontal microbiota, causing alterations in host–microorganism intercommunication that are sufficient to cause an inflammatory disease (18). Alternatively, dysbiosis could be considered as a sign that specific bacteria are involved in the etiology of the disease, which involves “true” periodontal pathogens and novel species that are either absent or hardly detectable in the healthy state. In 2020, the etiology of periodontitis was revised by a group of researchers who formulated the “Inflammation-Mediated-Polymicrobial-Emergence and Dysbiotic-Exacerbation” (IMPEDE) model (19). This theory focuses on the central role of inflammation in the development of a dysbiotic microbiome as a continuum from health to disease, consistent with the latest classification system of periodontal disease (20). However, the role of bacteria remains indispensable in deriving the transition from periodontal health to periodontitis. The pioneering work of Socransky et al. grouped periodontal bacteria in the biofilm into color-coded complexes based on certain criteria that define the actual involvement of each bacterium in the pathogenic process of periodontal disease (21). The leading member of the red complex is *Porphyromonas gingivalis*, which is pathogenic even when present at a low abundance in the microbiome, due to its diverse virulence capacities that induce dysbiosis (22). However, enigmatic roles of other bacteria could have profound effects on the pathogenesis of dysbiotic biofilms (23). Expanding this list by adding novel bacteria would greatly increase the knowledge of the complex composition of dysbiotic biofilms, improving diagnostic tools and aiding in developing new therapeutic strategies for periodontal disease (18).

In recent decades, newly discovered microorganisms such as members of the *phyla Bacteroidetes*, *Firmicutes*, *Proteobacteria*, *Saccharibacteria*, *Spirochaetes*, and *Synergistetes* have been constantly linked to the pathogenesis of periodontal disease (24, 25). Aruni et al. (26) have shed light on *Filifactor alocis* belonging to the *phylum Bacillota*, which is significantly associated with periodontitis (27). *F. alocis* exhibits the potential to be classified as a “true” periodontal pathogen due to its higher incidence in the periodontal pocket compared to healthy sites (24, 27). Several studies indicated increased prevalence of *F. alocis* with increasing probing pocket depth (PPD) and clinical attachment loss (CAL) (28, 29). In fact, subgingival plaque samples from deep periodontal pockets demonstrated that *F. alocis* was the third most common putative periodontal pathogen in cases of what was formerly known as aggressive periodontitis (30). Interestingly, the incidence of F. *alocis* was even higher than red complex bacteria, *Aggregatibacter actinomycetemcomitans*, and *Fusobacterium nucleatum* in sites with deteriorating clinical periodontal parameters (31). Additionally, the bacterial load of this bacterium was significantly reduced following periodontal treatment (25, 32). Furthermore, *F. alocis* is equipped with sets of virulence factors and mechanisms that could modulate neutrophil function (33), deactivate the complement system (34, 35), and survive in the high-oxidative-stress microenvironment of periodontal pockets. The latter feature could be genetically transferred to other periodontal bacteria such as *P. gingivalis*, thereby enhancing their ability to survive and persist in the periodontal lesions (36). Virulence factors belonging to the repeats-in-toxins (RTX) family typically expressed in Gram negative bacteria, particularly *A. actinomycetemcomitans*, and are responsible for modulating host's immune response (37). FtxA is a novel virulence factor that has been identified in up to 50% of *F. alocis* isolated from periodontitis cases (38, 39). Results from a cohort including Ghanaian adolescents showed that the bacterial load of *ftxA*-positive *F. alocis* has dramatically increased over the two years follow-up in sites with progressive loss of attachment and also demonstrated a synergistic relation with *A. actinomycetemcomitans* (39). Similar results were observed in another study conducted in Australia that also indicated increased prevalence of *ftxA*-positive *F. alocis* in subgingival plaque samples collected from sites with deep periodontal pockets and increased tissue destruction (28). However, available high-quality association/elimination studies supporting the role of *F. alocis* as a pathobiont directly involved in periodontal disease pathogenesis are still insufficient. Additionally, gap in data about the prevalence of this bacterium in certain population such as Middle Eastern is also evident.

Therefore, this work aimed to assess the prevalence and proportion of *F. alocis* in stage 3 and 4 periodontitis cases and assess its diagnostic potentials to discriminate between these stages.

## 2 Materials and methods

### 2.1 Study design, settings, and eligibility criteria

This cross-sectional study was conducted at the Department of Periodontics, College of Dentistry, University of Baghdad from December 2023 to July 2024. The ethical rules stated by the Declaration of Helsinki for conducting studies involving humans were followed by this study. The study protocol was first submitted to the Research Ethics Committee, College of Dentistry, University of Baghdad to obtain ethical approval (Ref: 858, Date: 3/12/2023). All patients received a thorough description about all aspects of the study and then were asked to sign a consent form before performing any clinical work.

The sample consisted of 50 periodontitis patients, 29 (58%) male and 21 (42%) female, with an average age of 45.6 ± 11.2 years (Table 1). Eligible patients were consecutively recruited based on the inclusion criteria, which mandated that all patients must be adults aged >18 years, affected by periodontitis, and not suffering of any systemic diseases. The case definition of periodontitis stated by the 2017 AAP/EFP classification of periodontal disease was followed. Accordingly, periodontitis cases were diagnosed when periodontal examination demonstrated CAL involving ≥2 non-adjacent teeth interdentally, which was confirmed by the presence of alveolar bone loss on radiographs. Periodontitis was also de-fined when oral/facial aspects of ≥2 non-adjacent teeth were concurrently affected by CAL ≥3 mm and a PPD of ≥4 mm (28). Any patients reporting a smoking habit, systemic disease such as diabetes mellitus, or pregnancy were not included. Additionally, those reporting a history of periodontal therapy or consuming antimicrobials/nonsteroidal anti-inflammatory drugs in the last 3 months were excluded as well.

**Table 1:** Demographic and clinical parameters of the study population.

Variables	
Sex (*n*, %)	
Male	29, 58%
Female	21, 42%
Total	50, 100%
Age (years)	45.6 ± 11.2
Stage (*n*, %)	
3	24, 48%
4	26, 52%
Grade (*n*, %)	
B	16, 32%
C	34, 68%
Clinical parameters^a^	
Whole mouth (*n* = 727)	
BOP%	56.3 ± 19.3
PI%	72.1 ± 16.0
PPD (mm)	5.2 ± 0.8
CAL (mm)	4.2 ± 1.6
Periodontal pockets/patient	14.5 ± 11.2
Pooled sites (*n* = 114)	
BOP%	0.8 ± 0.4
PI%	0.6 ± 0.5
PPD (mm)	5.8 ± 3.1
CAL (mm)	6.5 ± 2.5

BOP, bleeding on probing; PI, plaque index; PPD, probing pocket depth; CAL, clinical attachment loss.

^a^ Mean ± SD.

### 2.2 Measurement of periodontal parameters

Full-mouth periodontal charting, including bleeding on probing (BOP), PPD, and CAL, was conducted at six sites of each tooth, excluding wisdom teeth. The plaque index (PI) (40) was dichotomized into 0,1 based on the absence or presence of plaque following the application of a disclosing agent (Biofilm Discloser, EMS, Switzerland). The periodontal probe was inserted into the depth of pocket/sulcus to record PPD and CAL, using the gingival margin and cementoenamel junction as reference points, respectively. After removal of the probe, bleeding that was elicited spontaneously or after 20 s following probing was recorded as positive BOP. Radiographs were used to confirm the diagnosis and draw information about the stage and grade of periodontitis. The stage was defined according to radiographic bone loss, at the site with the worst CAL, relative to the root that was further modified by the number of teeth lost due to periodontitis (4). The grade was calculated by dividing the percentage of bone loss severity at the worst site by the age of the patient (4). All measurements were conducted by a calibrated examiner (R.H.A) using a UNC-15 probe. This examiner received calibration sessions from an expert periodontist before commencing the study. The results were discussed to resolve any sources of discrepancy, and the sessions were repeated until agreement between examiners was reached. This was confirmed when the interclass coefficient for PPD and CAL, rounded to the nearest millimeter, was >90% and the kappa test for categorical parameters was >80%.

### 2.3 Sampling subgingival dental plaque biofilm

The targeted collection sites were first isolated with cotton rolls, and any supragingival plaque and deposits were removed using a sterile curette. Then, a previously sterilized 35# absorbent paper point, with a 0.5 cm tip cut, was carefully inserted into the periodontal pocket along the tooth surface until minimal resistance was felt. The paper point was left for 15 s, and then it was removed and immediately placed into a 0.6 ml sterile centrifugal tube containing a bead solution (41, 42). The samples were collected from the sites with the deepest PPD in each sextant, pooled together (43) according to the pocket depth, and transported in an ice box for storage at −20°C until analysis.

### 2.4 Polymerase chain reaction

Detection of copy numbers in the subgingival biofilm samples for total bacterial and *F. alocis* loads was performed via quantitative polymerase chain reaction (qPCR). First, the DNA was extracted from the biofilm samples using the ABIOpureTM Total DNA kit (ABIOpure, USA), following the steps recommended by the manufacturer. Lyophilized primers 5′-3′ and probes were purchased from Macrogen (Republic of Korea) for Universal 16S RNA (F-GATTAGATACCCTGGTAGTCCAC, R-TACCTTGTTACGACTT) (44) and *F. alocis* (45) (F-ACCCTCAAGTTGCCA AAATTATTAT, R-TACTCCCTTTCTTCTGGTTAAATCT, P-FAM-TCGCTCTTTTTGCCGCCTCTCTTGC). The sequences of the primers and probe were blasted them against reference oral bacteria gene sequence in the database at the National Center for Biotechnology Information (NCBI; http://www.ncbi.nlm.nih.gov/tools/primerblast/index.cgi?LINK_LOC=BlastHome) to check their specificity. The blasting in this database was limited to gene sequence derived from human oral samples. The total reaction mix volume was 10 μl, prepared by adding 5 μl of SYBR Green Master Mix (Go Taq® qPCR Master Mix, Promega, USA) for Universal 16S RNA, while for *F. alocis* Master Mix with probe was used. The reaction mix was completed with forward, and reverse primers (0.5 μl each), 2.5 μl of nuclease-free water, and 1 μl of DNA. The qPCR program consisted of one initial denaturation cycle at 95°C (5 min), denaturation cycles (×40) at 95°C for 20 s and annealing between 55° and 60°C for 20 s for *F. alocis* and universal 16S, respectively. The standard curves were produced by serially diluting known concentrations of universal 16S and *F. alocis* from 15 × 10^9^ and 40 × 10^9^ copy μl^−1^ to 15 and 40 copy μl^−1^, respectively. The linear regression formula was obtained by plotting the known concentrations on the *x*-axis against cycle threshold (C*_t_*) on the y-axis. This equation was used to calculate the unknown concentrations for *F. alocis* and universal 16S in the samples (Supplementary Figure S1).

### 2.5 Statistical analysis

Results were expressed as means, standard deviations, frequencies, and percentages for continuous and categorical data. The latter were compared using the chi-squared test. Comparison of bacterial loads was performed using the Kruskal–Wallis test followed by Bonferroni's *post hoc* test for multiple comparisons. While Mann–Whitney test was used for comparing two groups. The diagnostic potential of the microbial biomarkers to discriminate the stage and grade of periodontitis was determined by using the receiver operating characteristic (ROC) curve and area under the curve (AUC). A simple linear regression model was used, considering the *F. alocis* load as a dependent variable and continuous outcomes (PPD and CAL) as independent variables to investigate the correlation between microbiological and clinical parameters. The significant difference threshold was set at *p*-values of less than 5%. All statistical assays were conducted using GraphPad Prism software (version 9.0, USA).

## 3 Results

### 3.1 Basic demographic and clinical characteristics

The periodontitis patients included in this study (*n* = 50) were predominately diagnosed with stage 4 and grade C periodontitis. In these patients, a total of 727 sites with different PPD, from shallow to deep, were recorded. Pooling the subgingival biofilm samples from these sites resulted in 114 samples that were used for microbiological analysis. The clinical parameters for the whole mouth and pooled sites are described in Table 1.

### 3.2 Comparisons of clinical periodontal parameters

Comparisons of clinical periodontal parameters according to stage and grade are shown in Table 2. Both PPD and CAL were significantly higher in stage 4 periodontitis than in stage 3 counterparts, whether the whole mouth or pooled sites were compared. According to the grade of periodontitis, PPD was significantly higher in the grade C group than grade B at the whole-mouth and pooled-sites levels, whereas CAL only showed significant differences between grades B and C at the pooled-site level. Out of the 114 pooled sites, the majority were in the maxilla, where anterior and posterior teeth collectively accounted for 55.3% (*n* = 63), while the rest of the sites (*n* = 51, 44.7%) were in mandibular teeth (Table 3).

**Table 2:** Comparisons of clinical parameters according to stage and grade of periodontitis.

Clinical parameters	Stage		*p*-value*	Grade		*p*-value*
	3	4		B	C	
Whole mouth (*n* = 727)						
BOP%	0.5 ± 0.2	0.6 ± 0.1	0.20	0.5 ± 0.1	0.6 ± 0.2	0.11
PI%	0.6 ± 0.1	0.7 ± 0.1	0.15	0.6 ± 0.2	0.8 ± 0.1	0.06
PPD (mm)	4.9 ± 0.3	5.5 ± 0.9	0.002	4.8 ± 0.4	5.4 ± 0.9	**0** **.** **03**
CAL (mm)	3.4 ± 0.7	4.9 ± 1.7	<0.001	3.7 ± 0.6	4.4 ± 1.8	0.12
Pooled sites (*n* = 114)						
BOP%	0.8 ± 0.3	0.9 ± 0.1	0.04	0.8 ± 0.4	0.9 ± 0.3	0.51
PI%	0.5 ± 0.4	0.6 ± 0.5	0.68	0.6 ± 0.4	0.6 ± 0.5	0.76
PPD (mm)	5.7 ± 1.5	7.0 ± 2.7	0.005	5.8 ± 1.3	6.9 ± 2.8	**0** **.** **02**
CAL (mm)	4.2 ± 1.5	6.8 ± 3.4	<0.001	4.4 ± 1.2	6.4 ± 3.5	**<0** **.** **001**

All data are presented as mean ± SD.

BOP, bleeding on probing; PI, plaque index; PPD, probing pocket depth; CAL, clinical attachment loss.

* Significant difference at *p* < 0.05 using unpaired *t*-test. Bold font indicates significant differences.

**Table 3:** Distribution of sites between maxillary and mandibular teeth.

Sextant	*n*, %
Maxillary anterior	31, 27.2%
Maxillary posterior	32, 28.1%
Mandibular anterior	22, 19.3%
Mandibular posterior	29, 25.4%
Total	114, 100%

### 3.3 Microbiological analyses

**Figure 1:**
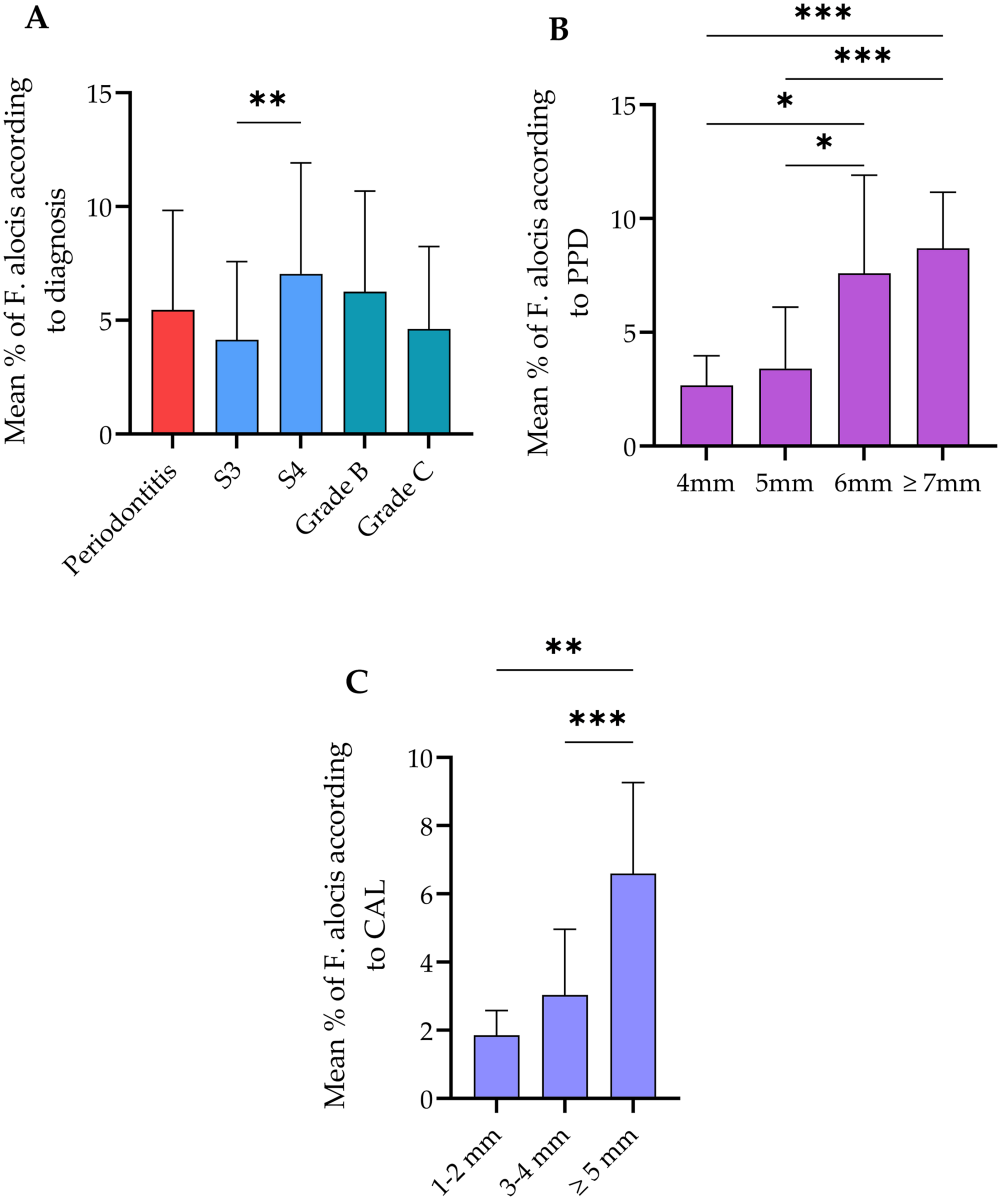
Relative abundance of *Filifactor alocis* in all cases and according to the **(A)** diagnosis statement, **(B)** probing pocket depth (PPD), and **(C)** clinical attachment loss (CAL). The total abundance of this bacterium was 6.3% relative to the total bacterial load. Stage 4 and grade C periodontitis exhibited significantly higher abundance of *F. alocis* compared to stage 3. No significant changes were observed between Grade B and C. **Significant difference at *p* < 0.002 usin Mann–Whitney test. Sites with PPD ≥6 mm showed significantly higher proportion of *F. alocis* in comparison with PPD between 4 and 5 mm. Sites with CAL ≥5 mm as harbored significantly higher *F. alocis* load than sites exhibiting lower CAL. *Significant difference at *p* < 0.01, **p < 0.002, ***p < 0.001 using Kruskal–Wallis test. Data expressed as mean ± SD.

Qualitative analysis of the relative abundance of *F. alocis* copies to the universal 16S RNA load is shown in Figure 1. In all included periodontitis cases, the abundance of *F. alocis* copies was 6.3% relative to the whole bacterial load in the subgingival microbiota. This abundance was significantly higher in samples collected from stage 4 periodontitis patients than stage 3 cases. No significant differences were detected according to grades of periodontitis. Additionally, the load of *F. alocis* was significantly increased in periodontal pockets exceeding 6 mm than sites with shallower PPD i.e., ≤5 mm. Data of bacterial load sorted according to pocket depth were clustered to the corresponding CAL measurements. The relative abundance of *F. alocis* in sites with CAL ≥5 mm was significantly higher than sites exhibiting 1–2 and 3–4 mm CAL.

Out of 114 subgingival biofilm samples, *F. alocis* was positively expressed in 78.9% of the sites. Analyses demonstrated no significant differences in frequency of sites with positive and negative expression of *F. alocis* when compared according to the stage, grade, PPD, and CAL (Table 4). Generally subgingival biofilm samples collected from periodontitis stage 4, and grade C showed higher frequency of *F. alocis* as compared to stage 3 and grade B cases. Increasing PPD and CAL were also associated with increased frequency of *F. alocis* in comparison with site exhibiting shallower PPD and less severe CAL.

**Table 4:** Positive and negative sites (*n* = 114) for *Filifactor alocis* expression according to the diagnosis domains, probing pocket depth (PPD), and clinical attachment loss (CAL).

Clinical parameters	*F. alocis* expression		*p*-value*
	Positive (*n*, %)	Negative (*n*, %)	
Total	90, 78.9%	24, 21.1%	
Stage			
3	36, 76.6%	11, 23.4%	0.64
4	54, 80.6%	13, 19.4%	
Grade			
B	30, 76.9%	9, 23.1%	0.80
C	60, 80.0%	15, 20.0%	
PPD			
4 mm	20, 74.1%	7, 25.9%	0.34
5 mm	23, 74.2%	8, 25.8%	
6 mm	19, 76.0%	6, 24.0%	
≥7 mm	28, 90.3%	3, 9.7%	
CAL			
1–2 mm	14, 66.7%	7, 33.3%	0.14
3–4 mm	27, 75.0%	9, 25.0%	
≥5 mm	49, 86.0%	8, 14.0%	

* Significant difference at *p* < 0.05 according to the chi-squared test.

**Figure 2:**
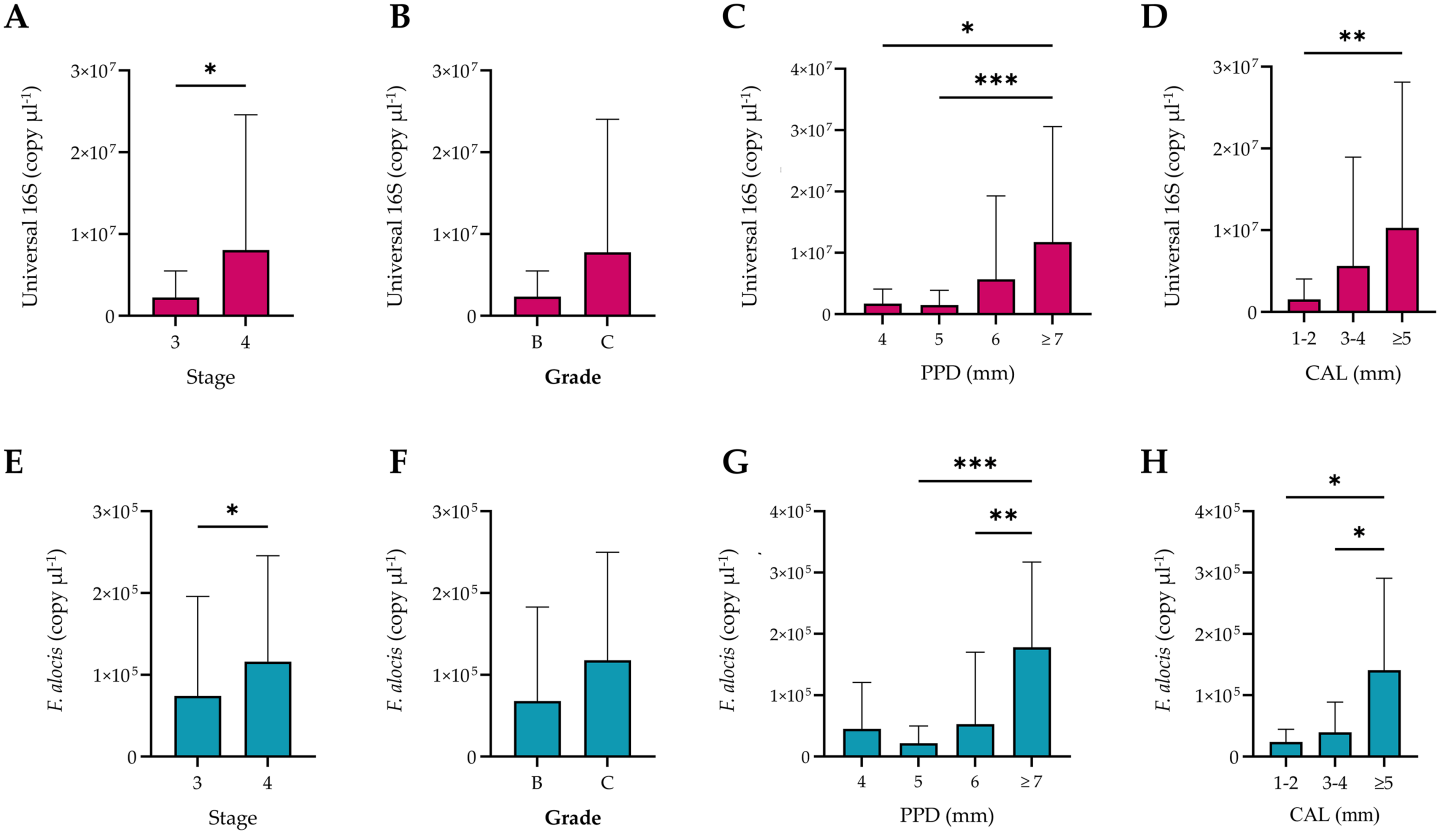
Quantification of the universal 16S RNA and *Filifactor alocis* copies according to stage, grade, probing pocket depth (PPD), and clinical attachment loss (CAL). The total bacterial load significantly increased with increasing severity of periodontitis **(A)**, PPD **(C)**, and severity of CAL **(D)**, but not the grade **(B)** Biofilm samples from stage 4 periodontitis, periodontal pockets ≥7 mm **(C)**, and CAL ≥5 mm **(D)** showed significantly higher copy numbers of *F. alocis* than other sites from stage 3 cases with shallower PPD and less severe CAL. Bars represent the mean, while error bars represent the standard deviation. Significant differences were considered at *p* < 0.05 using the Kruskal–Wallis test, where * *p* < 0.03, ** *p* < 0.002, and *** *p* < 0.001.

Quantitative PCR-based analyses of subgingival biofilm samples demonstrated a significant increase in the copy numbers for the total bacterial load with increasing severity i.e., stage, PPD, and CAL (Figure 2). However, no significant difference was observed when comparing grade B and C periodontitis. Regarding *F. alocis*, the same pattern was observed in association with the stage and grade of the disease. Additionally, periodontal pockets with depths of 7 mm or greater exhibited significantly higher copy numbers of this bacterium as compared to moderately deep periodontal pockets, which did not exhibit any significant differences among them. The same results were observed in association with sites exhibiting CAL ≥5 mm, which harbored significantly higher *F. alocis* loads than sites with less severe loss of attachment (Figure 2).

### 3.4 Diagnostic potential of the microbiological biomarkers

**Figure 3:**
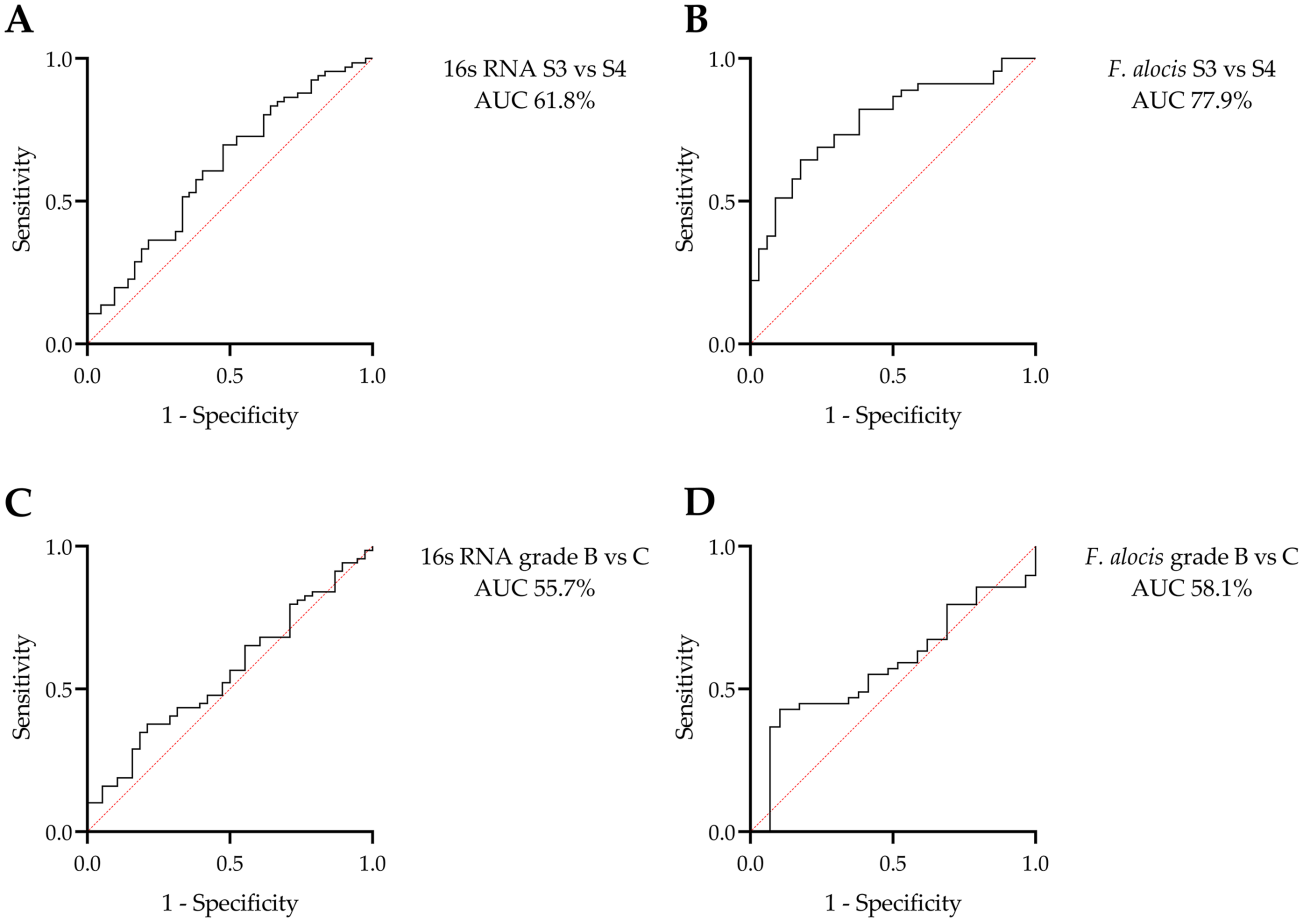
ROC analysis for the potential of the universal 16S RNA and *Filifactor alocis* copies to differentiate stages and grades of periodontitis: discrimination between stages (S) 3 and 4 using **(A)** universal 16S RNA and **(B)** *F. alocis* copies. Discrimination between grades B and C using **(C)** universal 16S RNA and **(D)** *F. alocis* copies. Only stages could be differentiated using these microbial biomarkers.

The diagnostic potential of universal 16S RNA and *F. alocis* to differentiate between stages and grades of periodontitis was explored using ROC analysis (Figure 3). Total bacterial load and *F. alocis* showed the ability to discriminate between stages 3 and 4 with moderate to good levels of accuracy (AUC 61.8% and 77.9%, respectively). However, neither biomarker reached a sufficient level of accuracy to differentiate between grade B and C cases. The proposed cutoff value of universal 16S RNA was 45,150 copies, with 66.7% sensitivity and 52.4% specificity, to differentiate stage 3 from stage 4 periodontitis, while the suggested cutoff value of *F. alocis* to differentiate these stages was 16,290 copies, with 82.2% sensitivity and 61.8% specificity (Table 5).

**Table 5:** AUC, sensitivity, specificity, and the proposed cutoff values for the universal 16S RNA and *Filifactor alocis* copies to differentiate between stages and grades of periodontitis.

Bacterial load	AUC	Sensitivity	Specificity	*p*-value	95% CI	Cut-off value
Universal 16S RNA						
S3 vs. S4	61.8%	66.7%	52.4%	**0** **.** **03**	0.508–0.727	45,150 copies
Grade B vs. C	55.7%	62.3%	44.7%	0.33	0.445–0.668	52,530 copies
*F. alocis*						
S3 vs. S4	77.9%	82.2%	61.8%	**<0** **.** **001**	0.677–0.880	16,290 copies
Grade B vs. C	58.1%	51.0%	58.6%	0.23	0.452–0.709	5,249 copies

AUC, area under the curve; CI, confidence interval; S, stage.

Bold font indicates significant differences.

Data from the simple linear regression model for continuous variables (Table 6) showed that the R^2^ values for PPD and CAL were 0.293 and 0.149, respectively. This meant that a one-unit increase in PPD or CAL (i.e., 1 mm) explained a 29.3% or 14.9% increase in the *F. alocis* concentration, respectively. This further suggested that pathological deepening of periodontal pockets and increasing severity of the disease were associated with increased populations of this bacterium. The same pattern was observed in association with variations in the universal 16S RNA concentration.

**Table 6:** Simple linear regression model for predictors of variation in total 16S RNA and *Filifactor alocis* concentrations (dependent variables).

Predictors	R^2^	Coefficients	SE	*p*-value	95% CI
Universal 16S RNA					
PPD	0.150	0.418	0.242	**<0** **.** **001**	5.650–6.611
CAL	0.092	0.303	0.310	**0** **.** **001**	4.838–6.069
*F. alocis*					
PPD	0.293	0.688	0.228	**<0** **.** **001**	2.413–4.699
CAL	0.149	0.179	0.314	**<0** **.** **001**	4.419–5.667

R^2^, coefficient of determination; SE, standard error; CI, confidence interval; PPD, probing pocket depth; CAL, clinical attachment loss.

Bold font indicates significant differences.

## 4 Discussion

In this study, *F. alocis* was positively expressed in 78.9% of the total samples, indicating its high prevalence in periodontal pockets. This was similar to what has been reported by other international studies for instance in Norway (87.5%), Switzerland (80%), Germany (66.7%) (30) and Korea (83%) (46). However, it was higher than prevalence found in Swedish (30%) (47) and Indian (53.3%) (48) populations which could be attributed to the impact of ethnic variations, and differences in assay used and sample size. Additionally, the abundance of *F. alocis*, relative to the total bacterial load in the subgingival biofilm samples, increased progressively with the stage of the disease and with increasing PPD and severity of CAL. This was further confirmed via regression analysis, which showed positive correlations between these clinical parameters and a steady increase in *F. alocis* concentration. The bacterial load of *F. alocis* was expressed at a higher level in stage 4 than stage 3 periodontitis, with the potential to discriminate between these stages. This bacterium has emerged in the last decade as a novel periodontal pathogen that could be included among Socransky's classical complexes, particularly the red complex. Previous association and elimination studies support the relevance of *F. alocis* to dysbiotic biofilms responsible for the initiation and progression of periodontal disease. However, the load of *F. alocis* in sites with different severities of periodontal disease was not fully elucidated according to the latest periodontal disease classification. Therefore, this study was designed and conducted to explore this aspect.

A microbial analysis is crucial when exploring subgingival microbiota associated with periodontal health and disease. Ideally, a site-specific microbial analysis would provide more detailed information; however, for practical and coast-related reasons, pooled techniques are used instead (49, 50). Additionally, pooled subgingival biofilm samples provide relatively high numbers of bacteria in comparison to site-specific samples (51). Pooling subgingival biofilm samples from selected or index teeth from sites exhibiting the same clinical condition, in order to investigate the microbiota, is a common practice that has been validated by several studies (41, 52–54).

Periodontitis-associated dysbiosis has been long attributed to a specific group of bacteria belonging to anaerobic Gram-negative taxa, such as *P. gingivalis*, *Fusobacterium nucleatum*, *Treponema denticola*, and *Aggregatibacter actinomycetemcomitans* (22, 55). The monopoly of these bacteria on theories explaining the pathogenesis of periodontitis has been broken by the introduction of advances in molecular and microbiological techniques, leading to the recognition of Gram-positive anaerobes in periodontal-disease-associated microbiota (56). In fact, commensal, and synergistic relationships between *F. alocis* and periodontal pathogens responsible for the destruction of periodontal tissues and architectural contributions to co-aggregation and maturation of the subgingival biofilm have been highlighted (30, 34, 36). Further evidence of the pathogenic features of *F. alocis* was derived from an *in vitro* study showing that the growth of *F. alocis* was reduced when co-cultured with *Streptococcus gordonii*, which is associated with a healthy periodontal microbiome (57). The subgingival microbiome of periodontal lesions with progressive CAL showed the co-existence of *F. alocis* and *Dialister pneumosintes*, and both bacteria showed the capacity to survive in periodontal pockets independent of other putative periodontal pathogens (48). This further highlights the possible pathogenic role of *F. alocis* during destructive events of periodontal tissues in the consortium of periodontitis. Resistance to oxidative stress is an outstanding virulence attribute of *F. alocis* that could significantly alter the dynamics of microbial communities (36, 58). This property can be transferred to other bacteria such as *P. gingivalis*, enhancing its survival up to 4-fold against high levels of oxidative stress in the inflammatory microenvironment (36, 59). Additionally, *F. alocis* can induce a range of inflammatory/immune responses that accelerate the rate of periodontal tissue breakdown, including apoptosis, impairing neutrophil extracellular trap production, and increased production of matrix metalloproteinase-1 and inflammatory cytokines (60–63). This was supported by the present study, which demonstrated a remarkable increase in the bacterial load of *F. alocis* in deeper pockets, mostly dominated by red-complex periodontal pathogens, and in sites with evidence of severe loss of attachment.

The available hierarchy of evidence supports the association of *F. alocis* with periodontal disease, with its prevalence significantly increasing in the subgingival microbiota of diseased sites as compared to its rare incidence at healthy sites (30, 48, 64–66). A previous report demonstrated that the prevalence of *F. alocis* in periodontitis patients from Australia was 82.5% (28), Germany, Norway, and Switzerland ranged between 66.7% and 87.5% (30), which were very close to its prevalence in Iraqi population (78.9%) in the current study. Additionally, Shaikh et al. (48) demonstrated that *F. alocis* in an Indian population was more prevalent (55.6%) at sites with a greater loss of attachment. This was consistent with our results, which also confirmed increased *F. alocis* concentration with increasing CAL. This finding was further supported by another longitudinal report that linked the concomitant presence of *A. actinomycetemcomitans* and *F. alocis* as an indicator for future bone loss (67). The increasing *F. alocis* prevalence in deep periodontal pockets observed in the present study was also indicated by Neelakandan et al. (65) and Schlafer et al. (30), who found increased *F. alocis* population with increased mean PPD (mainly 7–9 mm). Furthermore, negative presence of *F. alocis* at sites exhibiting shallow pockets or sites with no evidence of CAL vs. increased prevalence in moderately deep and deep periodontal pockets was demonstrated by a previous study (30).

Microbial biomarkers are valuable tools for predicting, diagnosing, and monitoring diseases characterized by the presence of multi-bacterial communities, such as periodontitis. Putative periodontal pathogens, associated with dysbiosis of dental biofilms, have shown promising ability to diagnose periodontal disease and predict outcomes of periodontal therapy with high accuracy (68, 69). ROC analysis conducted by Chen et al. demonstrated that *F. alocis* in salivary and dental plaque samples could differentiate individuals susceptible to periodontitis, with accuracy ranging between 73.9% and 81.7% (64). Schlafer et al. (30) also emphasized the diagnostic potential of *F. alocis*. This work indicated that both the total bacterial load and *F. alocis* could differentiate stages of periodontitis, but not the grade. This could be because the latter is a domain reflecting inherent biological information of the host, which anticipates the future progression of periodontitis, while the stage is a clinical indicator of disease severity.

Caution is advised when generalizing results of the present study, due to its small sample size. The lack of microbiological analyses of other putative periodontal pathogens was another limitation of this work. Such analyses are recommended in future studies to shed light on the possible interactions between these bacteria and *F. alocis*. Although this study aimed to investigate the prevalence and diagnostic power of *F. alocis*. for periodontitis stage 3 and 4, lack of healthy periodontium and milder periodontitis cases limited broader interpretations of the results. Additionally, site-specific, or salivary quantification of inflammatory cytokines could provide further understanding of the immune response aspects of this bacterium. Despite these limitations, the present study provides significant data that further confirm the association of *F. alocis* with periodontitis based on the latest classification of periodontal disease. Establishing a sufficient knowledge about bacterial biomarkers would greatly contribute to evidence-based dental practice by providing reliable diagnostic/prognostic tools for clinicians, particularly to compensate lack of experience when diagnosing cases in greyish zone and when conducting large-scale community-based surveys.

## 5 Conclusions

The results of this study demonstrated that the prevalence of *F. alocis* in periodontitis cases was considerably high (78.9%). The relative abundance of *F. alocis* in stage 4 was almost the double in stage 3 periodontitis, mainly at sites with deep periodontal pockets. Additionally, levels of this bacterium in subgingival microbiota possessed the potential to discriminate periodontitis cases with different severities that could be exploit as a diagnostic tool in clinical practice when current findings are confirmed by further studies.

## Data Availability

The raw data supporting the conclusions of this article will be made available by the authors, without undue reservation.

## Supplementary material

Supplementary Figure S1 Standard PCR curves for universal 16S RNA and *Filifactor alocis*.
