# Supplementary material for: Increased bacterial load of Filifactor alocis in deep periodontal pockets discriminate between periodontitis stage 3 and 4
Source: Front Oral Health. 2025 Mar 27;6:1543030. doi: 10.3389/froh.2025.1543030 (PMC11983625; doi:10.3389/froh.2025.1543030)
Supplement: Supplementary Figure S1 — Standard PCR curves for universal 16S RNA and Filifactor alocis. [file Datasheet1.pdf]

Absolute Quantification: Universal_16s

**Target**	Universal_16s → Universal_16s

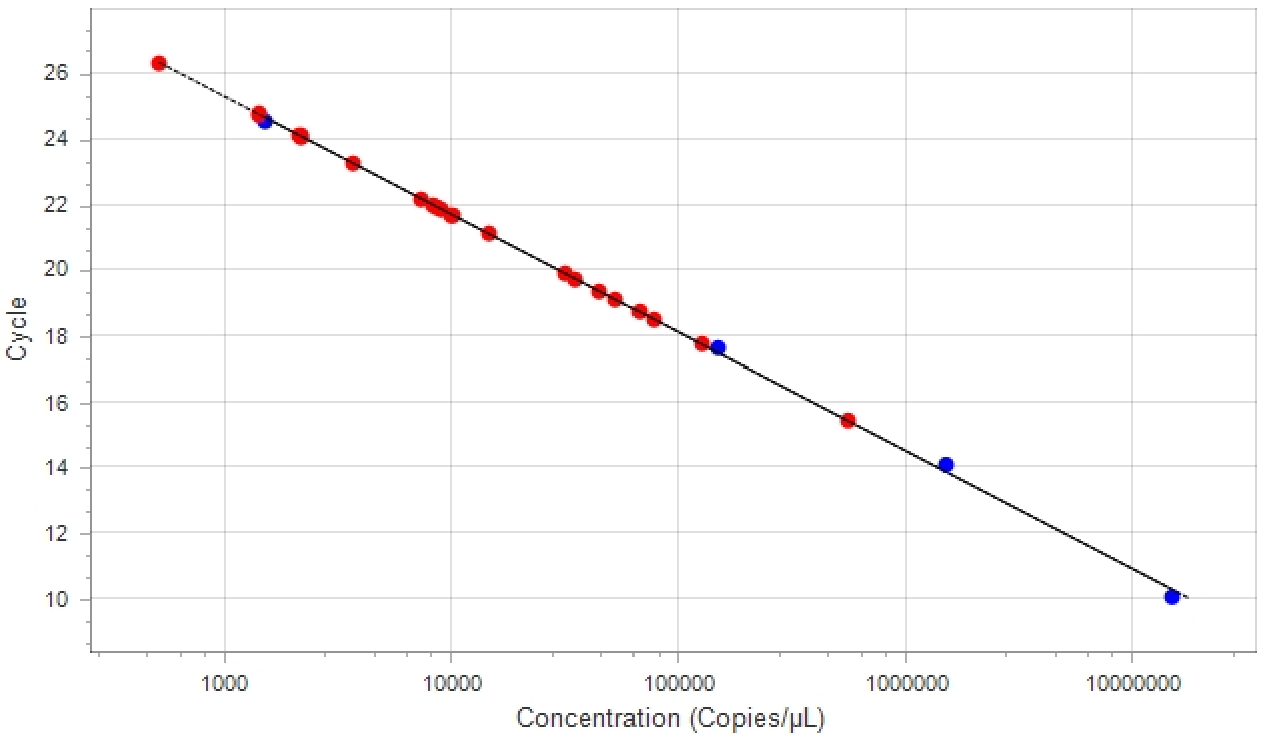

**Standard Curve**	
**Equation**	y = -3.60 x + 36.12
**Efficiency**	0.90
**R^2**	0.9986

Standards Results

Well	Sample Name	Cq	Given Concentration (Copies/μL)	Calculated Concentration (Copies/μL)	Variation
4[H]	SC4	10.03	1.5E+07	1.758E+07	17.18%
5[H]	SC5	14.09	1.5E+06	1.312E+06	12.51%
6[H]	SC6	17.66	1.5E+05	1.335E+05	11%
8[H]	SC8	24.54	1500	1644	9.6%

Absolute Quantification: F.alocis

**Target**	F.alocis → F.alocis

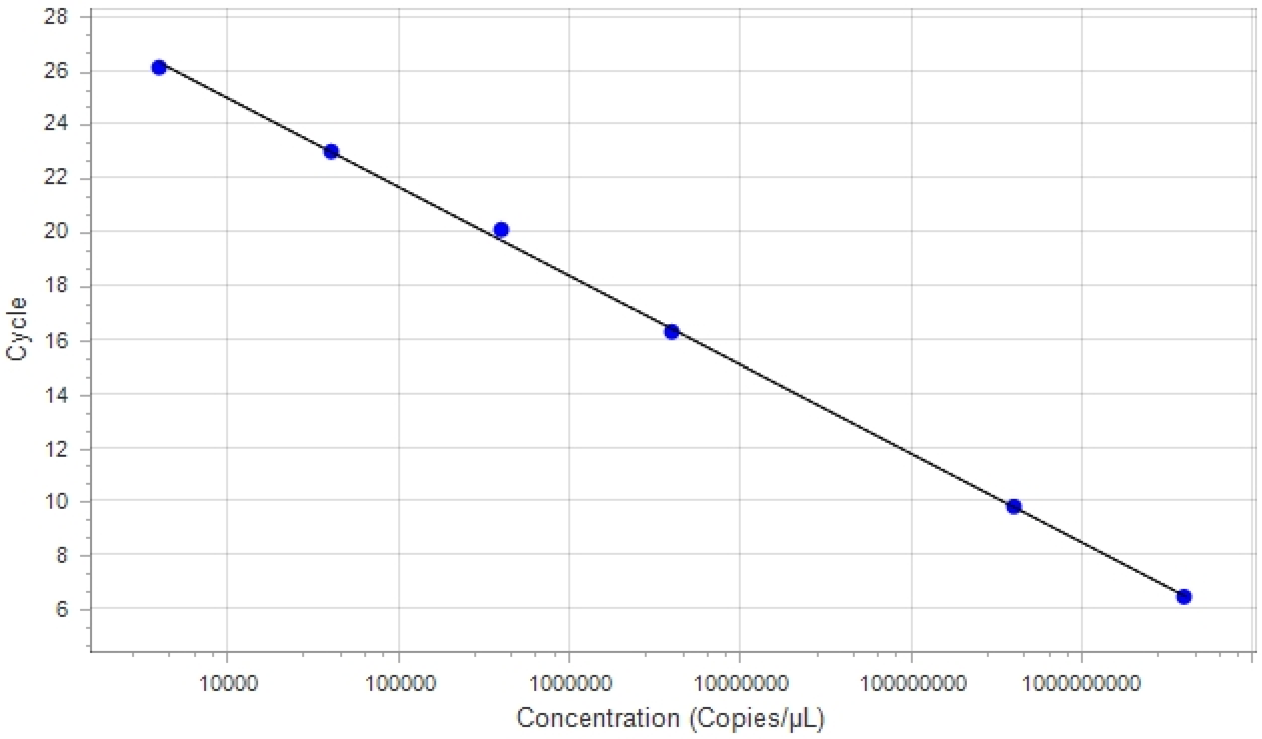

**Standard Curve**	
**Equation**	y = -3.30 x + 38.19
**Efficiency**	0.97
**R²**	0.9994

Standards Results

Well	Sample Name	Cq	Given Concentration (Copies/µL)	Calculated Concentration (Copies/µL)	Variation
1[B]	S1	6.43	4E+09	4.139E+09	3.48%
2[B]	S2	9.78	4E+08	3.999E+08	0.03%
4[B]	S4	16.25	4E+06	4.389E+06	9.72%
5[B]	S5	20.05	4E+05	3.111E+05	22.24%
6[B]	S6	22.96	4E+04	4.086E+04	2.14%
7[B]	S7	26.14	4000	4437	10.92%
